# Lumbosacral transitional vertebrae are associated with lumbar degeneration: retrospective evaluation of 3855 consecutive abdominal CT scans

**DOI:** 10.1007/s00330-020-06691-2

**Published:** 2020-02-19

**Authors:** Jaakko Hanhivaara, Juhani H. Määttä, Jaakko Niinimäki, Mika T. Nevalainen

**Affiliations:** 1 Department of Diagnostic Radiology, Oulu University Hospital, P.O. Box 50, 90029 Oulu, Finland; 2 Medical Research Center Oulu, University of Oulu, P.O. Box 8000, Oulu, Finland; 3 Research Unit of Medical Imaging, Physics and Technology, Faculty of Medicine, University of Oulu, POB 5000, FI-90014 Oulu, Finland

**Keywords:** Tomography, X-ray computed, Osteoarthritis, Facet joint, Spine

## Abstract

**Objectives:**

To assess the prevalence of lumbosacral transitional vertebra (LSTV) and associated spinal degenerative changes on abdominal CT scans in Caucasian population.

**Material and methods:**

A total of 3855 abdominal CT scans of the year 2017 from a single hospital were retrospectively assessed for LSTV, disc degeneration (DD), and facet joint degeneration (FD). An age- and sex-matched 150-subject control group without LSTV was picked at random. Multivariable logistic regression was used for the analysis.

**Results:**

LSTV was found in 1101 (29%) scans: Castellvi type I in 68%, type II in 16%, type III in 13%, and type IV in 3% of scans. Age- and sex-adjusted prevalence of DD was significantly higher in Castellvi type II and III groups at multiple lumbar levels, and in IV group at L4/5 than in control group (*p* < 0.001–0.034). At L5/S1, the prevalence of DD was significantly higher in the control group than in type II, III, or IV groups (*p* < 0.001–0.017). After combining Castellvi types II, III, and IV into one group, significant differences were found at all lumbar levels except L2/3 (*p* < 0.001–0.016). Prevalence of FD was significantly higher at L4/5 in Castellvi groups I, II, and III than in the control group (*p* < 0.001–0.002). When Castellvi types II, III, and IV were combined into one group, significant differences were found at lumbar levels L2/3, L3/4, and L4/5 (*p* < 0.001–0.021).

**Conclusion:**

Lumbosacral vertebrae of Castellvi types II, III, and IV are associated with greater lumbar degeneration, warranting meticulous evaluation of spinal anatomy, even on CT.

**Key Points:**

*• Lumbosacral transitional vertebra is a common incidental finding on abdominal CT scans with a high prevalence of 29%.*

*• When assessing whole lumbar spine, lumbosacral vertebrae of Castellvi types II, III, and IV were associated with greater lumbar degeneration, warranting careful evaluation of the lumbar spine on abdominal CT scans.*

## Introduction

Lumbosacral transitional vertebra (LSTV) is a common anatomical variant at the lumbosacral junction of the spine. The hallmark of an LSTV is the enlarged transverse process of the lowest lumbar vertebra. This transverse process can fuse to a varying degree with the adjacent ala of the sacrum either via pseudoarticulation or complete osseous fusion [1, 2]. The most commonly used classification for LSTVs is the Castellvi radiographic classification [3]. Previous studies have reported the prevalence of LSTV to be between 2.6 and 35.6% [4–8]. The wide range of prevalence may be due to heterogeneous evaluation of LSTV and imaging modalities [7, 9, 10]. French et al reported the prevalence of LSTV in the Australian population to be 9.9% on 5941 anteroposterior radiographs [7]. Another large-scale study using radiographs reported the prevalence of LSTV in Chinese Han population as 15.8% [8]. A recent study reported the prevalence of LSTV as 32% using MRI in young men population with low back pain [11].

LSTV has a controversial association with low back pain [1, 2]. The earliest accounts of symptomatic LSTV were reported by Bertolotti in 1917 [12]. The association of LSTV and low back pain has since been a topic of debate. Some authors report no association between LSTV and low back pain [13–15], whereas several studies suggest a positive association [8, 16, 17]. Accordingly, the presence of LSTV affects the distribution of degenerative changes in the spine. In patients with LSTV, degenerative changes occur more frequently at the level cranial to the LSTV, i.e., at the L4/5 level, whereas degeneration at the L5/S1 level is substantially less prevalent [14, 18–20]. It has also been reported that the degenerative changes occur at an earlier age than in patients without LSTV [14].

Traditionally, the presence of LSTV has been assessed on radiographs [1]. Several studies have also applied magnetic resonance imaging (MRI) [11, 14, 20] but studies using computerized tomography (CT) are very scarce [18]. As CT is considered to be the best imaging modality for the osseous anatomy, the aim of our study was to assess the prevalence of LSTV in the Caucasian population on abdominal CT scans. Furthermore, we evaluated the association of LSTV with spinal degenerative changes on every lumbar level and further detailed the classic Castellvi classification system.

## Material and methods

### Patients

Institutional review board approval was obtained and the requirement for informed consent was waived for this retrospective study. A PACS search for abdominal CT scans performed in the year 2017 at our institution was conducted. All abdominal CT scans were obtained regardless of the imaging indication, and there was no information about spine-related history. Scans not showing the lumbar spine fully and two patients on the basis of young age and immature skeletal development were excluded. Patients with spinal fusion implants were included in the evaluation of the prevalence of LSTV, but excluded from the analyses of degenerative changes. Accordingly, the search yielded 3855 CT scans. The study group’s median age was 65.3 years (ranging from 18 to 100 years), and 65% were male. Finally, an age- and sex-matched control group was selected at random, which consisted of 150 subjects without LSTV; the amount of controls was roughly matched to the median of LSTV subtypes.

### Image analysis

The 3855 CT scans were assessed for the presence of LSTV using Castellvi classification [3]. Type I includes unilateral (Ia) or bilateral (Ib) enlarged transverse processes measuring at least 19 mm craniocaudally; type II exhibits an enlarged transverse process with unilateral (IIa) or bilateral (IIb) pseudoarticulation with the adjacent sacral ala; type III describes unilateral (IIIa) or bilateral (IIIb) complete osseous fusion of the transverse process with the adjacent sacral ala; type IV represents a unilateral type II transition with a type III on the contralateral side [1]. The last ribs were used to define the Th12 vertebra, and the L5 vertebra was generally considered LSTV. The articulation of the transverse process was considered fused if an osseous bridge covering over 50% of the pseudoarticulation to the sacrum was present. We also came across subtypes not included in the original Castellvi classification: type IIa and IIIa LSTVs with enlarged contralateral transverse processes. We propose these new subtypes IIc and IIIc to be consistent additions to the Castellvi classification. Positive scans were evaluated for disc and facet joint degeneration (DD and FD, respectively). Since DD cannot be assessed directly on CT, we also evaluated the secondary end-plate changes. As no grading system for DD in the lumbar spine for CT exists [21], we used a modified radiographic grading system by Lane et al [22] for the evaluation of DD. The degree of degeneration was assessed at all lumbar levels and graded on a scale of 0 to 3, reflecting the following: 0, normal finding; 1, mild degeneration with minor osteophytes and/or mild narrowing of disc space; 2, moderate degeneration with distinct osteophytes, narrowing of disc space, and/or sclerosis; and 3, severe degeneration with large osteophytes, obliteration of disc space, sclerosis, and/or subchondral cysts. FD was evaluated using a similar four-tiered grading system by Weishaupt et al [23]. When grading the facet joints of the same lumbar level, the facet joint which exhibited a more severe degree of degeneration was chosen for grading. The initial imaging data was collected by a medical student and then re-evaluated by a radiologist with 5 years of experience.

### Imaging technique and statistical analysis

The slice thickness of the CT scans was 0.6 mm, which was applied for both the grading of LSTV anatomy and degenerative changes. Coronal, sagittal, and axial imaging planes were used for the analyses. If severe scoliosis existed, oblique multiplanar reconstructions were applied for the image interpretation.

In statistical analyses, both DD and FD grades were further dichotomized into two categories: grades 0 and 1 were classified as normal and grades 2 and 3 as degenerated discs or facet joints. Different Castellvi groups were first analyzed separately, and later Castellvi groups II, III, and IV were further combined into one pooled group in statistical analyses. Chi-square test was used to compare the groups (unilateral LSTV). Logistic regression analysis was used to assess the association of DD and FD between LSTV and the control groups. The inter-reader and intra-reader reliabilities for the Castellvi grading were assessed on 100 randomly picked subjects and analyzed using Cohen’s kappa (*κ*). The readers were a radiologist with 5 years of experience and a senior MSK fellowship-trained radiologist with 20 years of experience. As there was no referral or clinical data, the readers were not blinded. Statistical software (IBM SPSS Statistics for Windows, Version 24.0) was used for the analysis, and J.M. conducted the statistical analyses.

## Results

### Prevalence

Out of 3855 abdominal CT scans, LSTV anatomy was found in 1101 (28.6%) cases. Castellvi type I was found in 754 (68.4% of LSTV studies) studies, type II in 171 (15.5%) studies, type III in 143 (13.0%) studies, and type IV in 33 (3.0%) studies. Unilateral LSTV was more common on the left than on the right side (55.8% vs. 44.2%, respectively, *p* < 0.001). For the Castellvi grading, both the intra-rater and inter-rater agreements were excellent (*κ* = 0.928 and 0.855, respectively). When the laterality of the Castellvi anatomy was also taken into account, the intra-rater and inter-rater agreements were 0.845 and 0.772, respectively.

**Fig. 1:**
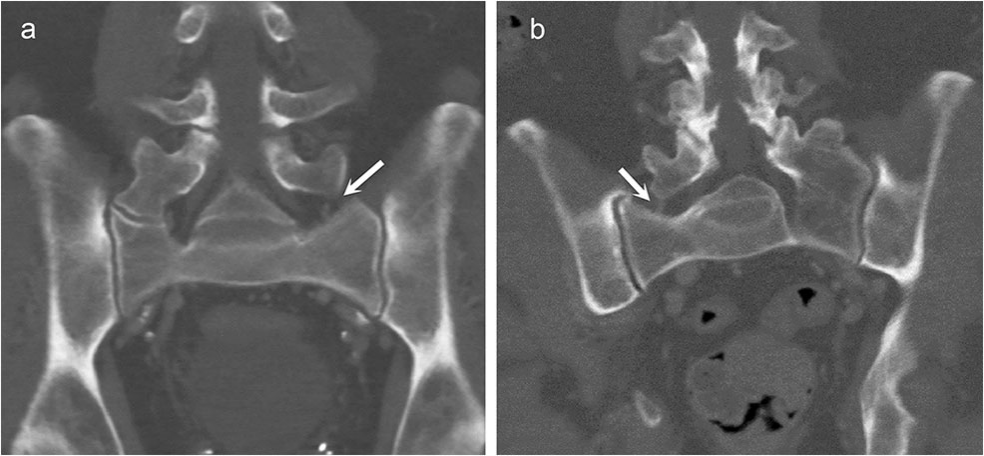
The proposed new subtypes to the classic Castellvi classification on coronal CT images. A 63-year-old male with suggested type IIc lumbosacral transitional vertebra (LSTV) showing a pseudoarticulation on the right side and an enlarged transverse process without articulation on the left side (arrow) (**a**). A 78-year-old male with suggested type IIIc LSTV demonstrating a complete fusion on the left side and an enlarged transverse process without articulation on the right side (arrow) (**b**)

Males and females had type I LSTV in 77% and 54% of all cases; type II 11% and 24%; type III 10% and 18%; and type IV 3% and 4%, respectively (*p* < 0.001). Additionally, type IIa and IIIa LSTVs with enlarged contralateral transverse process (height greater than 19 mm) were identified in 49 (4.5% of LSTV studies) and 9 (0.8%) studies, respectively. These types have not been included in the classic Castellvi classification during the modern era of imaging. Therefore, they are noted here as types IIc and IIIc as a logical continuum of the Castellvi classification as demonstrated in Fig. 1.

### Disc degeneration

**Fig. 2:**
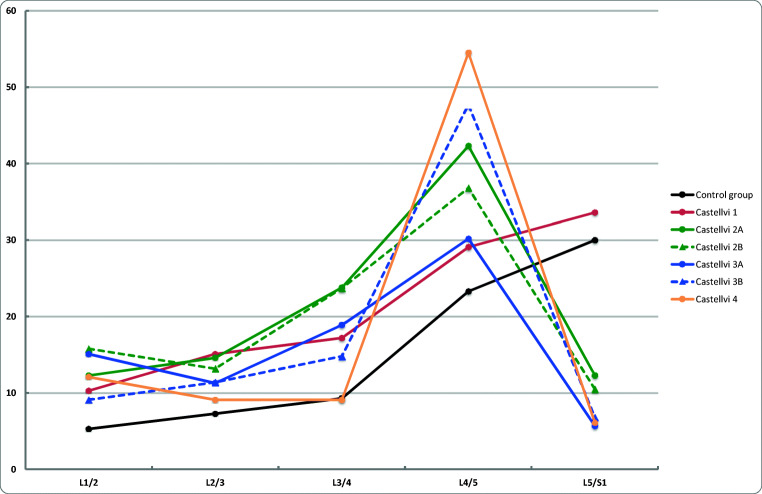
The prevalence of disc degeneration (in percentage) by disc levels in Castellvi groups and the control group

**Fig. 3:**
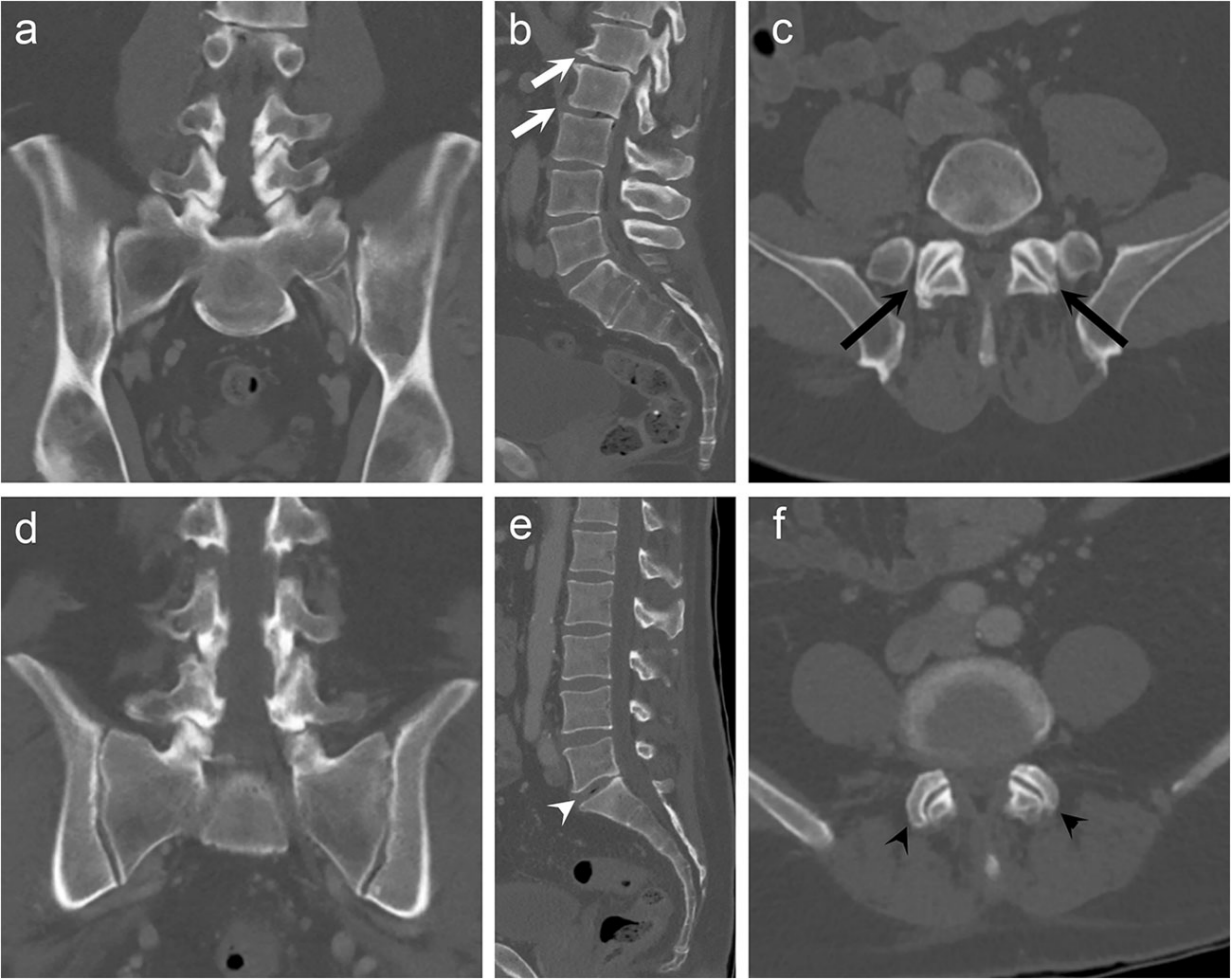
General lumbar degeneration is associated with lumbosacral transitional vertebra (LSTV). Coronal CT image (**a**) of a 64-year-old male with a type IV LSTV describing moderate degeneration at upper lumbar spine (white arrows) in sagittal view (**b**) and at L4/5 facet joints (black arrows) in axial view (**c**). Coronal CT image (**d**) of a 64-year-old male without LSTV showing only mild disc degeneration at L5/S1 (white arrowhead) in sagittal view (**e**) and at L4/5 facets (black arrowheads) in axial view (**f**)

The prevalence of DD in Castellvi groups and in the control group by disc level is shown in Fig. 2. After adjustments with age and sex, the prevalence of DD was significantly higher in Castellvi type II group than in the control group at lumbar levels L1/2, L3/4, and L4/5 (Table 1). With regard to type III, the prevalence of DD was higher at lumbar levels L3/4 and L4/5 than in the control group. In addition, the prevalence of DD was significantly higher at L1/2 and L4/5 in type IV group than in the control group. At L5/S1, the prevalence of DD was higher in the control group than in type II, III, or IV groups. When analyzing type I and control groups, the prevalence of DD was significantly higher in type I group only at L2/3. After combining Castellvi types II, III, and IV into one pooled group, significant differences were found at all lumbar levels except L2/3. Table 1 summarizes the distribution of disc degeneration by lumbar level in different Castellvi groups and controls. Figure 3 demonstrates an example of type IV LSTV with general lumbar degenerative changes and a control subject with less degeneration.

**Table 1:** Castellvi types by disc level that are significantly associated with higher disc degeneration vs controls

Castellvi type	Coefficient	Standard error	Wald chi^2^	*p* value	OR	95% CI
L1/2						
Type II	0.995	0.470	4.486	0.034	2.705	1.077–6.796
Type IV	1.658	0.766	4.680	0.031	5.247	1.169–23.560
Pooled	1.016	0.422	5.814	0.016	2.763	1.210–6.312
L2/3						
Type I	0.680	0.344	3.915	0.048	1.975	1.006–3.875
L3/4						
Type II	1.299	0.361	12.954	< 0.001	3.667	1.807–7.442
Type III	0.833	0.386	4.652	0.031	2.301	1.079–4.905
Pooled	1.004	0.329	9.342	0.002	2.730	1.434–5.200
L4/5						
Type II	1.060	0.283	13.981	< 0.001	2.886	1.656–5.031
Type III	0.983	0.280	12.350	< 0.001	2.672	1.544–4.623
Type IV	2.152	0.510	17.784	< 0.001	8.600	3.164–23.380
Pooled	1.112	0.244	20.724	< 0.001	3.040	1.884–4.906
L5/S1						
Type II	− 1.342	0.333	16.292	< 0.001	0.261	0.136–0.501
Type III	− 2.088	0.424	24.291	< 0.001	0.124	0.054–0.284
Type IV	− 1.879	0.790	5.660	0.017	0.153	0.033–0.718
Pooled	− 1.631	0.289	31.823	< 0.001	0.196	0.111–0.345

Binomial logistic regression analysis was performed using age, gender, and Castellvi type at every lumbar level. Pooled group is a combined group consisting of Castellvi type II, III, and IV groups

*CI* confidence interval, *OR* odds ratio

### Facet degeneration

**Fig. 4:**
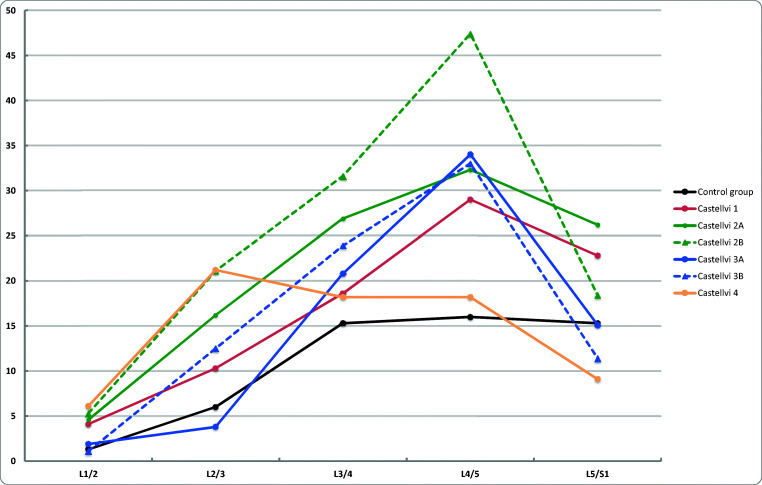
The prevalence of facet degeneration (in percentage) by disc levels in Castellvi groups and the control group

The prevalence of FD in Castellvi groups and in the control group by disc level is shown in Fig. 4. After adjustments with age and sex, the prevalence of FD was significantly higher at L4/5 in Castellvi groups I, II, and III than in the control group. In type II group, the prevalence of FD was additionally significantly higher at L2/3 and L3/4 than in the control group. In type IV group, the prevalence of FD was higher at level L2/3 than in the control group. Again, when Castellvi types II, III, and IV were combined into one pooled group, significant differences were found at lumbar levels L2/3, L3/4, and L4/5 (Table 2).

**Table 2:** Castellvi types by disc level that are significantly associated with higher facet joint degeneration

Castellvi type	Coefficient	Standard error	Wald chi^2^	*p* value	OR	95% CI
L2/3						
Type II	1.121	0.415	7.299	0.007	3.068	1.360–6.920
Type IV	1.824	0.604	9.108	0.003	6.195	1.895–20.252
Pooled	0.970	0.387	6.276	0.012	2.638	1.235–5.633
L3/4						
Type II	0.794	0.313	6.458	0.011	2.213	1.199–4.082
Pooled	0.653	0.282	5.355	0.021	1.922	1.105–3.342
L4/5						
Type I	0.799	0.257	9.655	0.002	2.222	1.343–3.678
Type II	1.132	0.303	13.967	< 0.001	3.101	1.713–5.613
Type III	1.028	0.311	10.917	0.001	2.795	1.519–5.142
Pooled	1.029	0.271	14.433	< 0.001	2.798	1.646–4.758

Binomial logistic regression analysis was performed using age, gender, and Castellvi type at every lumbar level. Pooled group is a combined group consisting of Castellvi type II, III, and IV groups

*CI* confidence interval, *OR* odds ratio

## Discussion

This is the first large-scale study to evaluate the prevalence of LSTV and to show the association of LSTV with whole lumbar degenerative changes using CT scans. The prevalence of LSTV was 28.6%. Castellvi types III and IV, and especially type II, had greater overall disc and facet degeneration in the lumbar spine compared with type I and the control group. We also observed type IIa and IIIa LSTVs with an enlarged contralateral transverse process and as these have not been described in the classic Castellvi classification, we noted these as types IIc and IIIc, respectively, for consistent addendum to the Castellvi classification.

Studies have usually evaluated the association of LSTV with degenerative changes only at the transitional and adjacent levels, showing accelerated degeneration at the level above and protective effect to the transitional level [11, 14, 20]. The rationale for this is that when one segment is stabilized, the other segment will have greater mobility and stress further leading to accelerated degeneration [20]. This is a similar phenomenon to lumbar stabilization studies where fusion has been associated with accelerated disc degeneration adjacent to fusion level [24, 25]. However, as all lumbar segments contribute to movements of the lumbar spine [26], in the authors’ opinion it is of interest to also assess the other lumbar segments than only the transitional and adjacent levels. In fact, we found LSTV and especially type II Castellvi group to have significantly greater degenerative changes also at the upper lumbar levels than the control group (Fig. 2, Fig. 3). Therefore, we suggest to consider also the upper lumbar levels when evaluating the patient’s clinical status and to assess these levels in the studies where one segment is stabilized.

It is reasonable that changes in L5/S1 level will affect the general structure and mobility of the lumbar spine. Vertebrae in LSTV are shown to be smaller and to have an altered facet morphology, too [27, 28]. Additionally, iliolumbar ligaments are weaker and thinner above the LSTV level [29]. As iliolumbar ligaments are important for torsional stability and in flexion-extension and lateral flexion movements [30, 31], it is plausible to state that those play at least a small role in association of LSTV with degenerative changes. Considering the L5/S1 level, osseous fusion or pseudoarticulation restricts movement and protects the L5/S1 level from degeneration [10].

Whether LSTV has any clinical relevance is under debate. The association of LSTV with low back pain has been described already in 1917 by Bertolotti [12]. There are multiple studies that show no association with any clinical symptoms and, on the contrary, numerous studies that show the association of low back pain with specific Castellvi types [1, 10]. The etiology of low back pain in cases with LSTV could originate from superjacent disk pathology, facet joint arthrosis, extraforaminal stenosis, or degeneration of the abnormal articulation of LSTV and sacrum [1]. When considering low back pain and imaging findings of the lumbar spine, DD has been found significantly more prevalent among subjects with back pain compared with asymptomatic subjects in a meta-analysis [32]. In this study, we evaluated disc degeneration of the whole lumbar spine and found that subjects with LSTV had greater lumbar spine degeneration. These novel findings help us to consider the clinical relevance of LSTV more thoroughly.

As CT offers superior resolution to assess bony contours, we noticed that some subjects had nearly fused LSTVs of type II. It appeared that some subjects had a congenital LSTV of type III, whereas some subjects had a LSTV of type III born out of degenerative osteophytic fusion. We also observed types IIc and IIIc which have not been noted in the classic Castellvi classification (Fig. 1). In our opinion, this is rationalized by the logic behind the classic Castellvi classification, as type I has an enlarged transverse process and IIa and IIIa have only either unilateral pseudoarticulation or complete osseous fusion [3]. In fact, the authors are relatively surprised that these subtypes have not been suggested earlier in the modern era of cross-sectional imaging.

Several limitations exist in this study. First, no clinical data was available on this study population, which prevents evaluation between low back symptoms and LSTV anatomy. Second, although CT gives good resolution of the bony structures, it can be limited in visualization of the articular surface of the transverse processes—especially with the type II LSTV. Third, the grading of type II and III LSTV posed issues, since in some cases partial coalition of the transverse process was observed; here, we classified the LSTV as type III if more than 50% of the articular surface was fused. Fourth, as no whole spine imaging was available, the counting of vertebrae presented some issues; consequently, the last ribs were used to define the Th12 vertebra. Moreover, we did not study the prevalence of sixth lumbar vertebra. When considering the vertebral numeration, the only reliable method has been found to be imaging of the whole sagittal spine [33]. Due to the nature of our study, we could not provide this data. However, as the sixth lumbar vertebra has not been found so prevalent [5], we think that lack of this information does not have a significant effect on our results.

In conclusion, LSTV is a common incidental finding on abdominal CT scans with a high prevalence of 29%.

We found that LSTVs of Castellvi types II, III, and IV are associated with greater lumbar degeneration, suggesting careful evaluation of spinal anatomy, even on CT scans.

## Abbreviations

CT: Computerized tomography
DD: Disc degeneration
FD: Facet joint degeneration
LSTV: Lumbosacral transitional vertebra
MRI: Magnetic resonance imaging
